# PncStress: a manually curated database of experimentally validated stress-responsive non-coding RNAs in plants

**DOI:** 10.1093/database/baaa001

**Published:** 2020-03-17

**Authors:** Wenyi Wu, Yan Wu, Dahui Hu, Yincong Zhou, Yanshi Hu, Yujie Chen, Ming Chen

**Affiliations:** 1 Department of Bioinformatics, State Key Laboratory of Plant Physiology and Biochemistry, College of Life Sciences, Zhejiang University, Hangzhou 310058, China; 2 College of Life Sciences and Food Engineering, Inner Mongolia University for the Nationalities, Tongliao 028043, China

## Abstract

Non-coding RNAs (ncRNAs) are recognized as key regulatory molecules in many biological processes. Accumulating evidence indicates that ncRNA-related mechanisms play important roles in plant stress responses. Although abundant plant stress-responsive ncRNAs have been identified, these experimentally validated results have not been gathered into a single public domain archive. Therefore, we established PncStress by curating experimentally validated stress-responsive ncRNAs in plants, including microRNAs, long non-coding RNAs and circular RNAs. The current version of PncStress contains 4227 entries from 114 plants covering 48 biotic and 91 abiotic stresses. For each entry, PncStress has biological information and network visualization. Serving as a manually curated database, PncStress will become a valuable resource in support of plant stress response research.

## Introduction

Plants growing in nature are constantly exposed to ever-changing environmental conditions including a wide array of abiotic and biotic stress stimuli. Such unpredictable stress conditions would make a severe impact on the growth and development of plants and even affect the yield of crops. For example, abiotic stress like temperature extremes, heat or cold, would result in the accelerated generation of reactive oxygen species, leading to oxidative stress damage, irreparable metabolic dysfunction and death (1,2). And various pathogens would challenge the host’s immune system, which is a major biotic threat other than insect pests (3). To survive and accommodate to external conditions, plants have evolved an elaborate defense system through specificity or cross-talk mechanisms based on transcriptional, post-transcriptional, translational and post-translational processes (4–6).

**Figure 1:**
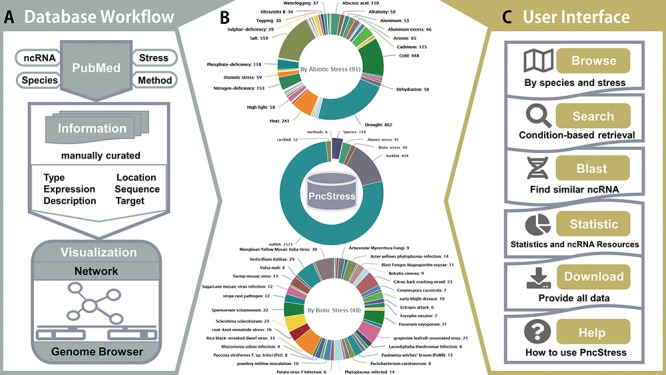
The architecture and statistics of PncStress.

With the popularity of the next-generation sequencing technologies and bioinformatics approaches, increasing research have shown that plant non-coding RNAs (ncRNAs) are widely involved in stress-responsive regulatory processes (7–9). ncRNAs constitute a class of RNAs that can function but not translated into proteins, including microRNAs (miRNAs), long non-coding RNAs (lncRNAs) and circular RNAs (circRNAs) (10–14). These types have been confirmed to play important roles in plant stress responses (1,15–19). miRNAs have emerged as vital regulatory molecules in plants and animals (20,21). Plant miRNAs and their regulatory targets are involved in plant responses to the environment. There is evidence that the expression of miR395 increases while its target ATP sulfurylase 1 (APS1) declines upon sulfate starvation in *Arabidopsis thaliana* (22). Studies illustrate miR169-mediated regulation is critical for adaptation to drought stress in *A. thaliana* (23), *Oryza sativa* (24) and *Medicago truncatula* (25). Other experiments have confirmed that ta-siRNAs show down-regulation in drought and high-salinity stress in *A. thaliana*, which are derived from TAS ncRNAs and targeted by miRNA (26). Recent studies also show that lncRNAs perform biological roles in various development processes. One of the typical examples is the regulation exerted by cold-induced long antisense intragenic RNA and cold-assisted intronic noncoding RNA transcripts, which are initiated from the FLOWERING LOCUS C antisense transcripts that are repressed by prolonged cold (27,28). Hidden treasure 1 (HID1) has been proved to act as a positive lncRNA regulator of photomorphogenesis in continuous red light (29). Under dehydration, phosphate deficiency and heat stress, circRNAs have been reported to act as sponges of miRNAs (30).

Due to the expanding research efforts on plant ncRNAs by high-throughput and low-throughput techniques, a large number of ncRNA candidates have been deposited. Examples include PNRD (31), NONCODE (32) and lncRNAdb (33). However, the existing databases have not systematically curated stress-responsive ncRNA in plants that have been experimentally validated by low-throughput experiments. Therefore, we manually curated 2523 miRNAs, 444 lncRNAs and 52 circRNAs in PncStress, which have been validated to respond to 139 stresses in 114 plants. We provide each entry with specific information gathered from the literature, such as stress, species, expression pattern, experimental method, functional description, genomic position and sequence. PncStress is the most comprehensive database after comparing with PASmiR (34), lncRNAdb (33), EVLncRNAs (35) and circFunBase (36). For those published databases and tools, we provide an integrated interface based on different research fields.

**Figure 2:**
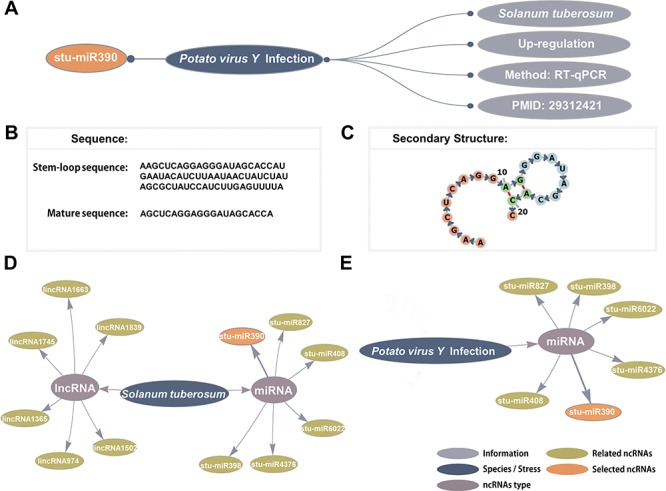
Detailed information and network visualization. (A) The basic information of stu-miR408. (B) The stem-loop sequence and mature sequence of stu-miR390. (C) The secondary structure of stu-miR390. (D) The *S. tuberosum*–ncRNAs network. (E) The *Potato virus Y* infection–ncRNAs network.

## Materials and Methods

### Data compilation

The collection of all PncStress entries was performed as follows. We firstly filtered out articles related to miRNA, lncRNA and circRNA from PubMed that were connected with stress, response, defense and adaptation. Considering the ncRNA alternate name, we used a list of keywords containing all the synonyms during the query. Take the term `lncRNA’ as an example, `lncRNA’, `long noncoding RNA’, `long non-coding RNA’ or `long non-protein coding RNA’ were extensively searched. Then both scientific and generic names of plants were added as keywords to filter the results, like `*Vitis vinifera’* and `Grape’, `*Zea may’* and `maize’. From these literatures, we manually curated ncRNAs and its biological information confirmed by low-throughput methods [e.g. reverse transcription-polymerase chain reaction (RT-PCR), quantitative RT-PCR, stem-loop RT-PCR or northern blot]. For more functional information, we predicted the secondary structure by RNAfold (37), interaction candidates by psRNATarget (38) and miRNA-triggered phasiRNAs by PhaseTank (39).

### Database construction

The PncStress database was implemented using HTML and PHP. MySQL was used for data storage and efficient management. Bootstrap and JavaScript libraries jQuery were used to make the interface application and network visualization. Dalliance (40) was used to view the genome, in which the genomic context of ncRNAs was based on the annotation from Phytozome (41). The BLAST module was implemented using SequenceServer (42).

**Figure 3:**
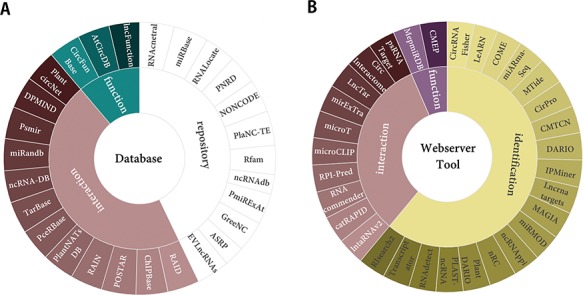
Plant ncRNAs research resources. (A) The 27 popular databases of ncRNAs. (B) The 31 useful webservers and tools of ncRNAs.

## Results

### Database architecture and data statistics

Figure 1 shows the database architecture of PncStress. All entries are processed through the `Data Workflow’ (Figure 1A). Each validated ncRNA contains manually curated information from PubMed, including stress, species, expression pattern, experimental method, genomic position, concrete sequence, functional description, target gene and reference literature. The visualization consists of networks and genome browser. `User Interface’ provides browsing, search, statistics and downloads as well as online BLAST service (Figure 1C). Each section contains documentation to ensure effective use.

The statistics of PncStress are summarized in three parts (Figure 1B). In the current version, PncStress possesses 4227 entries, including 2523 miRNAs, 444 lncRNAs and 52 circRNAs in response to 48 biotic stresses and 91 abiotic stresses, which have been validated by six experimental methods in 114 species. The statistics also show the number of each abiotic and biotic stress-responsive ncRNAs. Drought, salt, cold and heat are the most abundant stresses with 862, 559, 448 and 241 associated ncRNAs, respectively.

### Data retrieval

PncStress provides a three-part retrieval system. Firstly, users can retrieve all stress-responsive ncRNAs by species or stresses in the `Browse’ page. Species are sorted in alphabetical order, while stresses are divided into biotic and abiotic. Users can find information of every items from each result table, such as species name, stress name, expression pattern, validated method and PMID, which are also part of network visualization in the `Detail’ page (Figure 2A). The `Detail’ page contains additional information of each ncRNA, including the location, sequence, validated target, secondary structure, genome browser, functional description and interaction candidates. In addition, stem-loop sequence and mature sequence from miRBase (43), predicted alignment files predicted by psRNATarget and miRNA triggered phasiRNAs predicted by PhaseTank are provided special for miRNAs. As an example, the sequence and secondary structure of stu-miR390 are displayed in Figure 2B and C.

Secondly, we constructed species–ncRNAs and stress–ncRNAs networks that directly retrieve stress-responsive ncRNAs in each species and stresses. Six miRNAs and six lncRNAs respond to *Solanum tuberosum* (Figure 2D), while six miRNAs respond to *Potato virus Y* infection (Figure 2E). Both networks are associated with stu-miR390. The researchers found up-regulation of miR408, miR827, miR398 and miR390 in potato cultivar Désirée, which is tolerant to *Potato virus Y* infection as well as down-regulation of miR4376 and miR6022 (44). Users can click the interested node to its detailed page.

In addition, PncStress provides four advanced search options to retrieve data and enable auto-completion and fuzzy search for the text fields. Users can search by species, stress, ncRNA name and three ncRNA types, or combine two or more options.

### Additional ncRNA resources

Given the more practical requirements in plant ncRNAs research field, PncStress provides a collection interface of the most popular and topic-specific ncRNA resources in the `Statistics’ page. The 58 web resources were grouped into categories of identification, function, interaction and repository, including RNAcentral (45), NONCODE (32), PceRBase (46) and MTide (47). They were divided into two parts: 27 databases and 31 webservers/tools (Figure 3). Users can hover over the desired item to get its description and click to access its web page.

## Discussion

Plant stress-responsive biomolecules have attracted increasing attention, and the involvement of ncRNAs has expanded to further understanding plant stress response mechanisms. Thus, obtaining experimentally validated stress-responsive ncRNAs directly from a large number of studies and their biological information would become a problem for individual researchers. Here, we have developed PncStress, a repository that manually curated the three most crucial stress-responsive ncRNAs in plants. PncStress provides detailed page with tables and visualization for users to obtain stress-responsive ncRNAs information. Take stu-miR390 for example, it was up-regulated in response to *Potato virus Y* infection in *S. tuberosum*, which has been validated through RT-qPCR in a study that was comprised in PubMed with a unique identifier (PMID 29312421). This information can be retrieved from the table and is part of network visualization in the `Detail’ page. For other ncRNAs that respond to several stress conditions, we divided their stress conditions into biotic and abiotic. Stu-miR390 associated with other miRNAs and lncRNAs in *S. tuberosum*–ncRNAs network and *Potato virus Y* infection–ncRNAs network. The intertwining of different networks indicates the crucial regulatory role of ncRNAs in plant survival and adaption to external conditions. We also have constructed ncRNA-related networks for other plants and stresses. Additional manually curated information from PubMed are displayed for each validated ncRNA. Those predicted interaction candidates can serve as functional annotation to gain more insight into ncRNA regulation.

PncStress contains most of the plants and stress conditions. For instance, in *A. thaliana*, 305 miRNAs, 186 lncRNAs and 5 circRNAs were deposited in PncStress, which are experimentally validated to be associated with 26 abiotic stresses and 4 biotic stresses. PncStress is expected to be a fundamental study resource that includes data on the most important crops such as maize, wheat, cotton and potato.

In summary, PncStress is a manually curated knowledgebase of experimentally validated stress-responsive miRNAs, lncRNAs and circRNAs in plants. It will bridge the gap in ncRNAs functional research and further facilitate biologists in revealing the roles of ncRNAs in diverse stress conditions. We will update PncStress regularly based on newly published data. The improvement of PncStress can facilitate future plant study in stress responses.

## Funding

National Key Research and Development Program of China (nos. 2018YFC0310602 and 2016YFA0501704); National Natural Science Foundation of China (nos. 31771477 and 31571366); the Fundamental Research Funds for the Central Universities; Jiangsu Collaborative Innovation Center for Modern Crop Production and Collaborative Innovation Center for Modern Crop Production co-sponsored by province and ministry.

## Notes

**Database URL**: http://bis.zju.edu.cn/pncstress
